# St. Andrew’s Hospital for Mental Diseases, Northampton

**Published:** 1882-04

**Authors:** 


ST. ANDREW'S HOSPITAL FOR MENTAL DISEASES, NORTHAMPTON.—Case of Recurrent Melancholia, with Persistent Suicidal Attempts, characterized by the swallowing of large sharp-pointed bonnet pins and quilting needles; recovery; discharge. (Under the care of Mr. J. Bayley.)

For the following notes we are indebted to Mr. T. V. deDenne:

Miss ———, aged thirty-one, was admitted October 17, 1879, suffering from melancholia. This was the second attack, and had existed two months. She had been under treatment elsewhere four years previously. She was described in "the statement" as having talked of committing suicide, and as being dangerous to others "only when restrained from attempts to escape."

On admission the patient was somewhat depressed, and expressed a wish to destroy herself. During the night it was found that she had tied the cord of a window-blind round her neck. After the first few days, in which she constantly asked for poison, she became more cheerful, although varying from time time, and either laboring under a delusion that she would ruin the place if detained or speaking of suicide.

By December 6th she was apparently so much improved that she was allowed to accompany a skating party to a neighboring meadow which was flooded, when, successfully evading those around, she threw herself into a hole in the ice, from which she was rescued with some difficulty.

On January 12th, delusions and suicidal intent meanwhile persisting, she complained of pains in the abdomen, and vomited a fluid with a red paint-like sediment, but denied having taken anything of an unusual nature. After this she continued sick, vomiting green matter, and on January 25th, being then in considerable abdominal pain, chiefly on the left side, confessed that prior to the attempt to drown herself, she had swallowed bonnet pins, and broken crochet and knitting needles. The seat of pain was now transferred from the left to the right side of the abdomen, and continued until February 21st, when she vomited a lady's bonnet pin, three inches and a half in length, without the black head, and sharp at the point. The point of the pin stuck into the palate and had to be removed. Relief was immediate, but the following day she again began to vomit, and a hard, painful spot was observed to the right of the umbilicus. A day later the motions were found to be tinged with blood, and there were restlessness, passing into delirium, and tympanitis. On February 29, being a little more than a week from the appearance of the first pin, she passed another by the bowels of a similar kind with the head intact, and a few days afterwards two portions of knitting needles, of a larger size than the others, but of the same length.

Some time elapsed, during which she showed no mental improvement, and was more or less constipated and tympanitic.

On April 22d she passed with some pain and blood another portion of a knitting needle, and next day still another. This was the sixth and proved to be the last. Her mental condition was most variable; at one time she talked sensibly, and then she would laugh and express a wish to die, etc.

Subsequently an improvement was observable, and on September 23, 1880, being upwards of eleven months from the date of her admission, the patient was discharged recovered.

The treatment of the local symptoms at first consisted in the administration from time to time of castor-oil until the confession of the patient established their true origin, when special diet was ordered and an occasional enema was given, the tympanites, etc., being also, so far as possible, combated by the application of poultices or fomentations. Morphia was prescribed, after admission for the mental condition, but later chloral was the principal agent employed to allay restlessness and procure sleep.

*Remarks.*—The case is of interest as showing the length of time that foreign bodies may be retained in the intestinal canal. The first of these made its appearance about eleven weeks, and the last nineteen or twenty from the date given by the patient as that on which they were swallowed. And bearing in mind their dangerous nature, it is still more noteworthy for the satisfactory result.—*Lancet.*

